# Apoptotic efficacy of biogenic silver nanoparticles on human breast cancer MCF-7 cell lines

**DOI:** 10.1007/s40204-015-0042-2

**Published:** 2015-10-23

**Authors:** Jannathul Firdhouse M., Lalitha P.

**Affiliations:** Department of Chemistry, Avinashilingam Institute for Home Science and Higher Education for Women University, Coimbatore, 641043 Tamil Nadu India

**Keywords:** Nanosilver, Cytotoxicity, *Alternanthera sessilis*, MCF-7 cell line

## Abstract

This article describes the synthesis of silver nanoparticles using the aqueous extract of *Alternanthera sessilis* as a reducing agent by sonication, espousing green chemistry principles. Biologically synthesized nanoparticle-based drug delivery systems have significant potential in the field of biopharmaceutics due to its smaller size entailing high surface area and synergistic effects of embedded biomolecules. In the present work the cytotoxic effect of biosynthesized silver nanoparticles studied by MTT assay against breast cancer cells (MCF-7 cell line) showed significant cytotoxic activity with IC_50_ value 3.04 μg/mL compared to that of standard cisplatin. The superior activity of the silver nanoparticles may be due to the spherical shape and smaller particle size 10–30 nm as confirmed from transmission electron microscope (TEM) analysis. The data obtained in the study reveal the potent therapeutic value of biogenic silver nanoparticles and the scope for further development of anticancer drugs.

## Introduction

Infection paves a pathway to non-communicable diseases such as cardiovascular disease and cancer. Cancer is a multifaceted genetic disease caused primarily by environmental factors and its treatment is usually a combination of numerous varied modalities. Different types of cancers can behave very differently. Among these lung cancer and breast cancer are very disparate diseases. Breast cancer is a malignant tumor that starts in the breast cells and occurs particularly in women (Alison 2001). The Times of India (12 Oct. 2012) estimates 1,00,000–1,25,000 new breast cancer cases in India every year. This statistical number is estimated to double by 2025. The mortality rate of patients and the must for cancer therapy coerces the need for technological breakthroughs in terms of easy availability, cost effectiveness and safety in terms of side effects. Paclitaxel, an FDA approved breast cancer drug is used widely in breast cancer treatments. It is alleged to have a string of side effects. These stipulate the need for herbal medicines in cancer therapy.

Nanomedicine is an upcoming field that could potentially make a major impact on human health (Teli et al. 2010). Nanoparticles possess unique chemical, physical and biological properties, and hence it finds use in various fields like business, therapeutics, electronics, cosmetics, catalysis and drug delivery (Sriram et al. 2012). It offers a new view for tumor detection, prevention and treatment. Nanoparticles eradicate cancer cells by flow and penetration to different regions of tumors through blood vessels and then to interstitial space to arrive at the target cells. The environmental and physiological characteristics vary from one tumor tissues to another. Hence nanoparticles should be designed in such manner, taking into account the target site and route of administration to generate optimal therapeutic effects (Wang et al. 2014).

Among nanoparticles, silver nanoparticles have an eye-catching role owing to their innumerable physical and chemical properties. Silver nanoparticles are more potent than the silver ions as revealed in avant-garde research. Silver nanoparticles are potential anticancer agents (Raghunandan et al. 2011). Cytotoxicity studies of silver nanoparticles using plant extracts: *Melia dubia*—human breast cancer cell line (Kathiravan et al. 2014), *Malus domestica* (apple) extract—MCF7 (Lokina et al. 2014), *Inonotus obliquus* (Chaga mushroom) extract—A549 human lung cancer (CCL185) and MCF7 human breast cancer (HTB22) cell lines (Nagajyothi et al. 2014), *Erythrina indica*—breast and lung cancer cell lines (Rathi Sre et al. 2015), *Piper longum* fruit—breast cancer cell lines (Reddy et al. 2014), *Annona squamosa* and *Brassica Oleracea.* var. *botrytis*—MCF-7 (Vivek et al. 2012; Ranjitham et al. 2013) are reported.

Silver nanoparticles synthesized using *Acalypha indica* Linn shows only 40 % cell inhibition against human breast cancer cells (MDA-MB-231) (Krishnaraj et al. 2014). The MCF-7 cells lose their 50 % viability with AgNPs (5 µg/mL) produced by *Dendrophthoe falcata* (Sathishkumar et al. 2014). *Datura inoxia* AgNPs inhibits 50 % proliferation of human breast cancer cell line MCF7 at 20 μg/mL after 24 h incubation by suppressing its growth, arresting the cell cycle phases, reducing DNA synthesis to induce apoptosis (Gajendran et al. 2014). Nuclear condensation, cell shrinkage and fragmentation are noticed for MCF-7 cells treated with *Sesbania grandiflora* mediated AgNPs (20 µg/mL) after 48 h in Hoechst staining.

*Morinda citrifolia* root extract-mediated AgNPs (100 µg) produced 100 % death of HeLa cell lines (Suman et al. 2013). Longer exposures to *Eucalyptus chapmaniana* AgNPs (0.02 mmol/mL) resulted in 85 % cell death after 24 h incubation (Sulaiman et al. 2013a, b). The viability of HL-60 cells decreased to 44 % after 6 h treatment with *Rosmarinus officinalis* AgNPs at 2 mM and cell death increased to 80 % after 24 h incubation (Sulaiman et al. 2013a, b). Cytotoxic activity was extremely sensitive to the size of the nanoparticles produced using *Iresine herbstii* leaf and the viability measurements decreased with increasing dosage (25–300 µg/mL) against the HeLa cell lines (Dipankar and Murugan 2012). *Piper longum*-mediated silver nanoparticles exhibit a significant cytotoxic effect (94.02 %) at 500 µg/mL on HEp-2 cell lines (Jacob et al. 2012). The therapeutic effect of silver nanoparticles may elicit through manipulation of their size, shape, elemental composition, charge and surface modification or functionalisation, leading target particles to specific organs (Thorley and Tetley 2013).

Owing to the significance of silver nanoparticles in cancer treatment and the necessity for newer breast cancer drugs, the present work spotlights the cytotoxic potential of the synthesized biogenic silver nanoparticles. Amid the cropping methods of nanosynthesis, biogenic synthesis finds healthier application in pharmacology due to the non-toxic nature of the source of capping material used.

*Alternanthera sessilis* is a weed growing on a variety of soil types. Its young shoots and leaves are ingested as vegetables. Phytochemical screening reveals the presence of reducing sugars, steroids, terpenoids, saponins, tannins and flavonoids in *A. sessilis* (Sahithi et al. 2011). The herb possesses antioxidant (Borah et al. 2011), anti-inflammatory (Sahithi et al. 2011), antipyretic (Nayak et al. 2010), haematinic (Arollado and Osi 2010), hepatoprotective (Lin et al. 1994), antiulcer (Purkayastha and Nath 2006), antimicrobial (Jalalpure et al. 2008), diuretic (Roy and Saraf 2008) and cytotoxic (Balasuriya and Dharmaratne 2007) activities. The herb is also reported as febrifuge, galactagogue, abortifacient, and used in the treatment of indigestion (Anandkumar and Sachidanand 2001). The plant is reported to contain lupeol, α and β-spinasterol, β-sitosterol, stigmasterol, campesterol, handianol, 24-methylenecycloartanol, cycloeucalenol and 5α-stigmasta-7-enol (Jou et al. 1979; Sinha et al. 1984).

High levels of ellagic acid and rutin are reported in the HPLC analysis of the ethanolic extract of *A. sessilis* (Mondal et al. 2015). Ellagic acid possesses a selective antiproliferative activity and induces apoptosis in Caco-2 colon, MCF-7, Hs 578T and DU145 cancer cells (Losso et al. 2004). Ellagic acid down-regulates the 17β-estradiol-induced hTERT α + β + mRNA expression and exerts chemopreventive effects in breast cancer (Strati et al. 2009).

With the aforesaid background necessitating research in newer breast cancer drugs, the present work is aimed at assessing the anticancer potential of plant-mediated silver nanoparticles in vitro, against human breast cancer cell lines MCF-7.

## Materials and methods

### Plant-mediated silver nanoparticles

Fresh aerial parts of *A. sessilis* were used to produce silver nanoparticles from silver nitrate. The aqueous extract of *A. sessilis* was treated with of silver nitrate (3 mM) solution (1:10) and sonicated using ultrasonic bath {Ultrasonics [1.5 L (H)]}. The optimized conditions for the formation of silver nanoparticles are reported in our earlier paper (Firdhouse and Lalitha 2013). The nanosilver formed was purified by repeated centrifugation and characterized.

### Characterization of silver nanoparticles

The nanoparticle formation was ascertained by recording UV–visible spectra (double beam spectrophotometer 2202- Systronics). The morphology and the particle size of the *A. sessilis* extract-mediated silver nanoparticles were characterized by transmission electron microscopy (FEI’s TecnaiTM G2 transmission electron microscope (TEM)).

### In vitro cytotoxicity study of silver nanoparticles

#### Cell culture

Human breast cancer cell line (MCF-7) was purchased from National Centre for Cell Science (NCCS), Pune. The cancer cells were grown in Eagle’s minimum essential medium (EMEM) containing 10 % fetal bovine serum (FBS) and maintained at 37 °C, 5 % CO_2_, 95 % air and 100 % relative humidity.

#### Cell treatment procedure

The monolayer cells were detached with trypsin–ethylene diamine tetra acetic acid (EDTA) to make single cell suspensions. The viable cells were counted using a hemocytometer. The cell solution was diluted with medium containing 5 % FBS to give final density of 1 × 10^5^ cells/mL. The cell suspensions (100 µL/well) were seeded onto 96-well plates, maintaining the plating density as 10,000 cells/well and incubated at 37 °C, 5 % CO_2_, 95 % air and 100 % relative humidity for cell attachment to the bottom of the wells. After 24 h, the cells were treated with serial concentrations of the nanosilver samples.

The nanosilver samples were passed through a 0.45-µm filter syringe. An aliquot (100 µL) of the sample solution was diluted to 1 mL with serum free medium. Twofold serial dilutions were made to provide a total of five sample concentrations. Varying concentrations (1.56, 3.12, 6.25, 12.5, 25 µL/mL) of silver nanoparticles were inoculated into grown cell containing 100 µL medium. Then the plates were incubated for 48 h at 37 °C, 5 % CO_2_, 95 % air and 100 % relative humidity. Varying concentrations (0.1, 1, 10, 50, 100 µM) of cisplatin were used as standard. The study was run in triplicate to ensure accuracy of the results.

#### MTT assay

The yellow solution of 3-[4,5-dimethylthiazol-2-yl] 2,5-diphenyltetrazolium bromide (MTT) (15 µL) was added to phosphate buffered saline (5 mg/mL) in each well. The plates were incubated at 37 °C for 4 h for the reduction of MTT. The resulting purple formazan crystals were solubilized in 100 µL of DMSO and the absorbance was measured at 570 nm using a micro plate reader (EMR500-Labomed). The cell inhibition (%) was calculated using the formula:

$$ {\text{Cell inhibition }}(\% ) = 100 - \left[ {{{{\text{Abs}}\left( {\text{sample}} \right)} \mathord{\left/ {\vphantom {{{\text{Abs}}\left( {\text{sample}} \right)} {{\text{Abs}}\left( {\text{control}} \right)}}} \right. \kern-0pt} {{\text{Abs}}\left( {\text{control}} \right)}}} \right] \times 100. $$

A non-linear regression graph was plotted between cell inhibition (%) and log_10_ concentration and the 50 % minimum inhibitory concentration (IC_50_) was determined using Graph Pad Prism software.

## Results and discussion

**Fig. 1:**
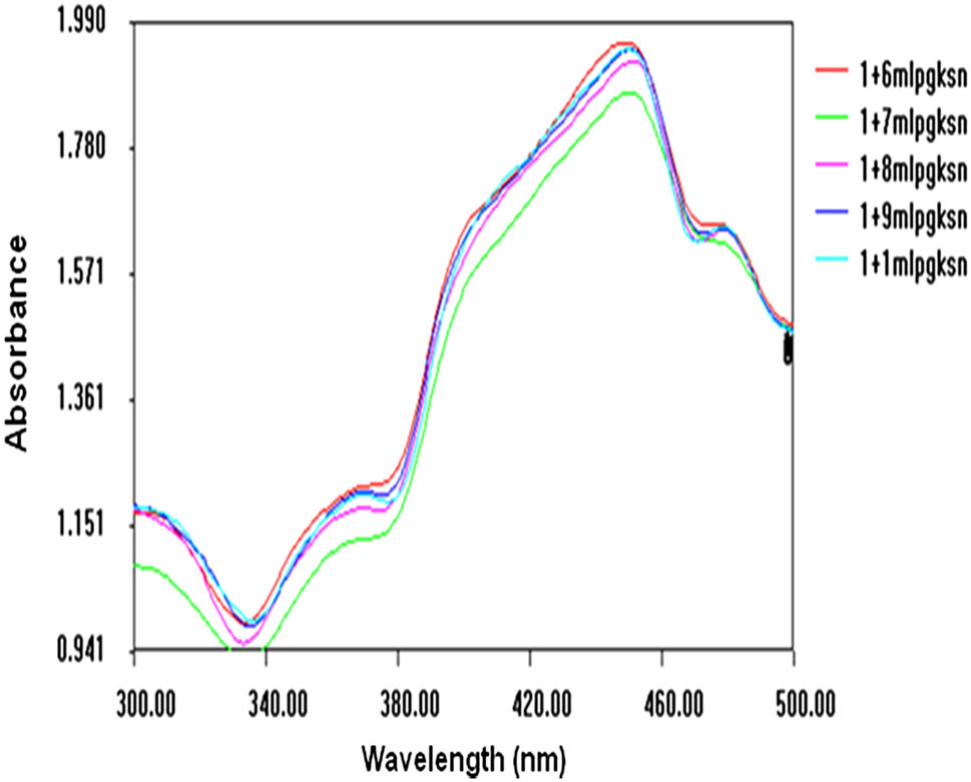
UV–visible spectrum of *Alternanthera sessilis* extract-mediated silver nanoparticles

**Fig. 2:**
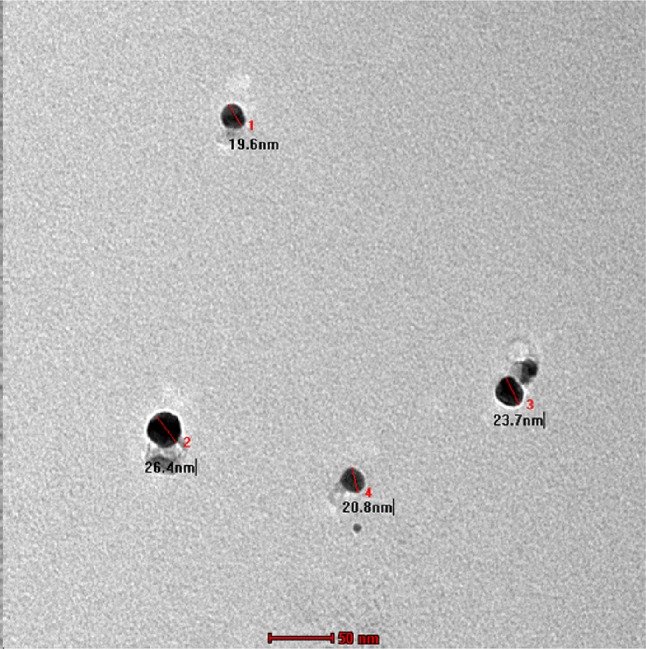
TEM micrograph of the biogenically synthesized silver nanoparticles

The fresh aqueous extract of *A. sessilis* and silver nitrate solution was mixed in 1:10 ratio and sonicated for 45 min. The yellow color solution changed to reddish-brown indicating the formation of silver nanoparticles. The completion of reduction of silver ions to nanosilver is evidenced from the broad surface plasmon resonance (SPR) band (420–450 nm) in the UV–visible spectra (Fig. 1). TEM analysis revealed the spherical morphology of the nanosilver with particle size in the range 10–30 nm (Fig. 2).

**Fig. 3:**
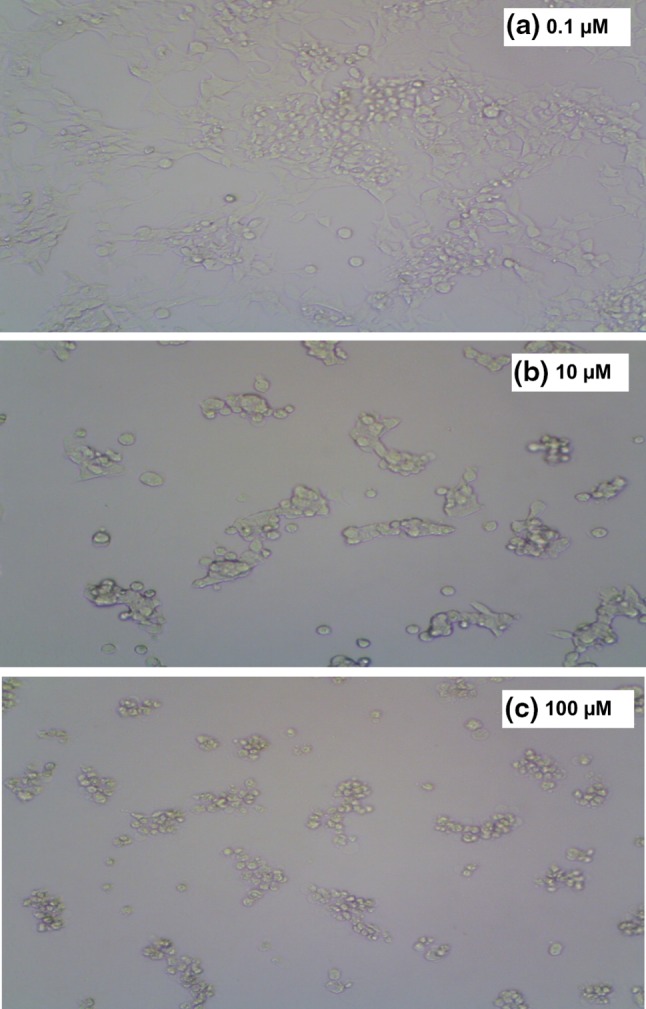
Cytomorphological changes and growth inhibition of cisplatin on MCF-7 cell line at **a** 0.1 µM, **b** 10 µM and **c** 100 µM concentrations

**Fig. 4:**
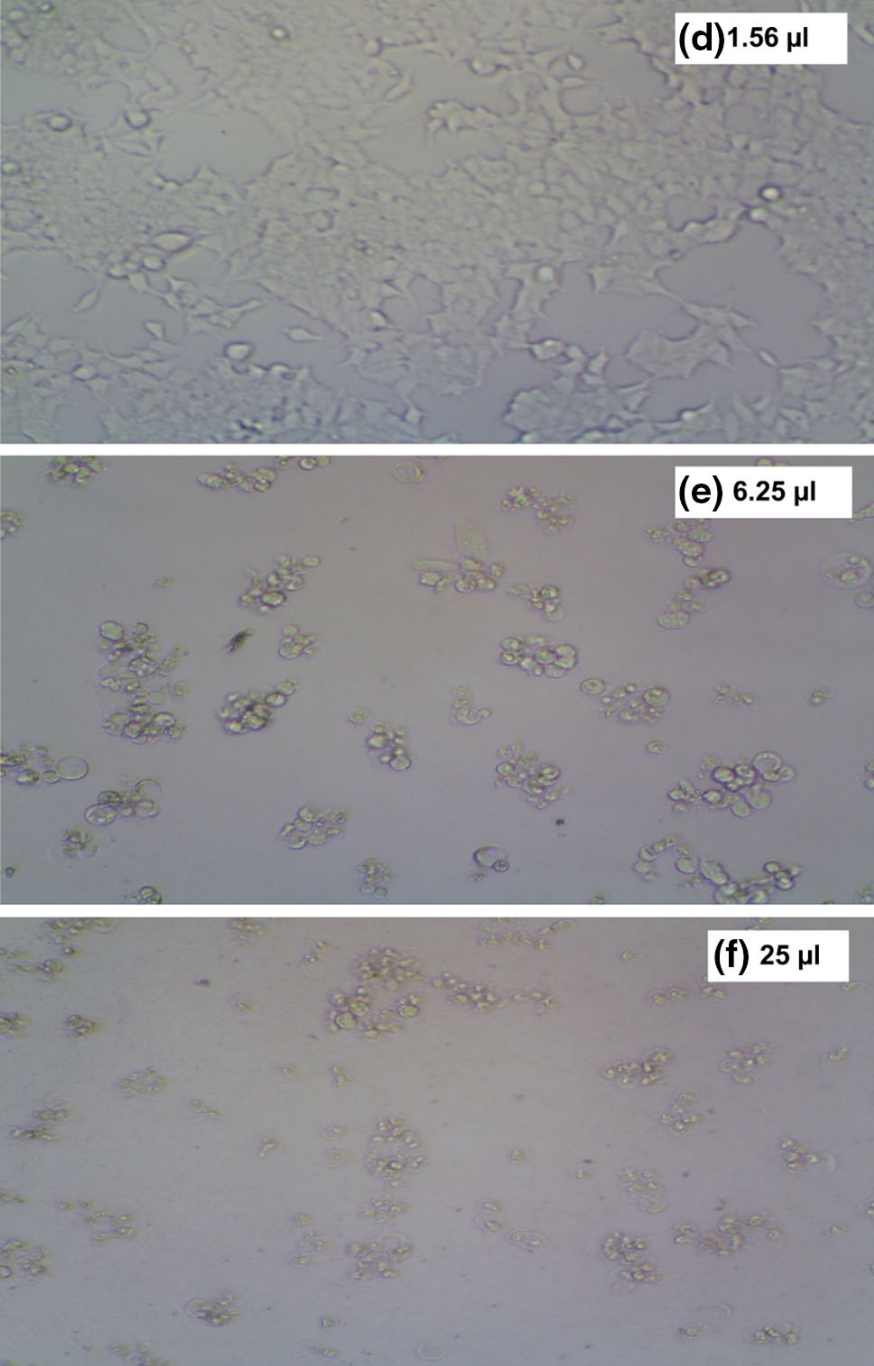
Cytomorphological changes and growth inhibition of silver nanoparticles on MCF-7 cell line at **d** 1.56 µL, **e** 6.25 µL and **f** 25 µL concentrations

**Fig. 5:**
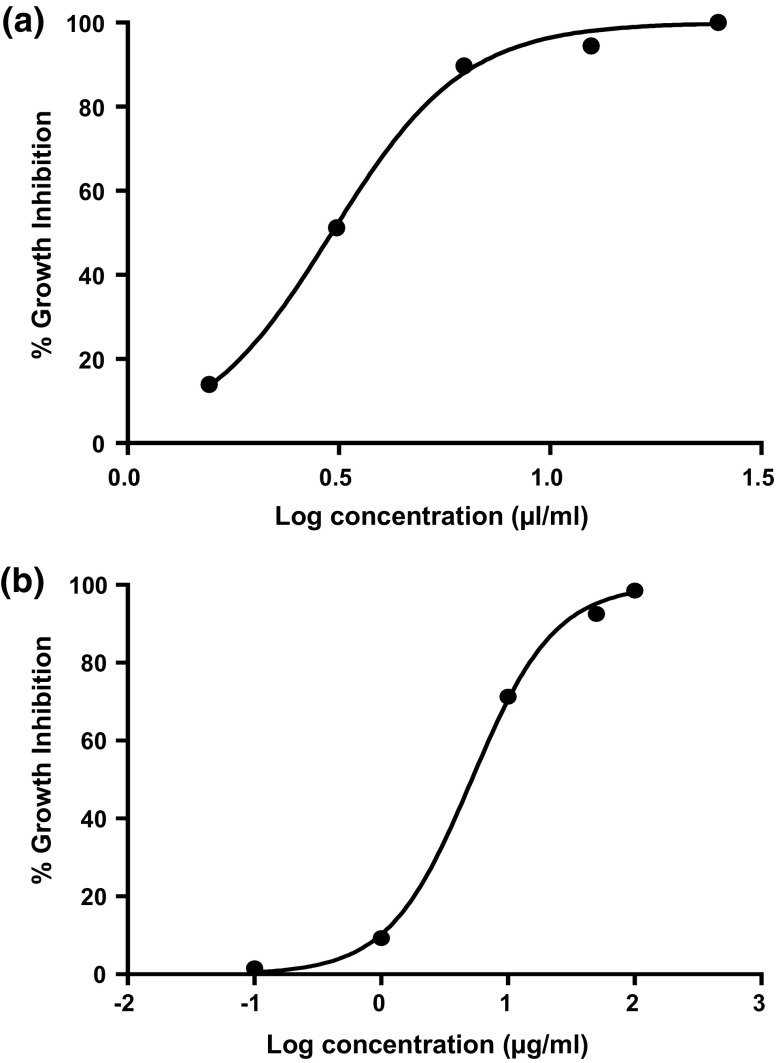
Cytotoxicity of *Alternanthera sessilis*-embedded silver nanoparticles (**a**) and cisplatin (**b**) on human breast cancer MCF-7 cell lines

The in vitro cytotoxic effect of silver nanoparticles against breast cancer cell lines (MCF-7) and cell inhibition (%) was carried out by MTT assay and compared with the standard cisplatin. Cisplatin, the commercially available anticancer drug, was used as standard and its cytotoxicity is shown in Fig. 3a–c. Comparison of the cytotoxicity of synthesized AgNPs (1.56, 3.12, 6.25, 12.5, 25 µL/mL) and cisplatin (0.1, 1, 10, 50, 100 µM) disclosed similar mortality rate. The cytomorphological changes of AgNPs on MCF-7 cell lines at different concentrations (1.56, 6.25, 25 µL) involve intracellular suicide program possessing morphological changes like cell shrinkage, oxidative stress, coiling and biochemical response leading to apoptosis as shown in Fig. 4d–f. It is quite obvious from the results that the apoptosis rate of MCF-7 cell lines increases with increase in concentration of silver nanoparticles (Fig. 5a). A dose-dependent increase in cell inhibition is seen after 48 h exposure The IC_50_ of cell inhibition of silver nanoparticles was observed at 3.043 µL/mL. The complete cell inhibition (99 %) of breast cancer cell lines was obtained at a maximum concentration of 25 µg/mL. These results evidence the dose- and time-dependent increase in cytotoxicity. The IC_50_ value predicts that the plant-mediated nanosilver proves to be a promising drug for chemotherapeutic treatment. Complete apoptosis was observed with 25 µg/mL of AgNPs, whereas it is 30 µg/mL (or 100 µM) for cisplatin.

*Alternanthera sessilis* is enriched with flavonoids, glycosides, sugars, amino acids and steroids. Flavonoids are polyphenols which exhibit a wide variety of biological activities such as antioxidant, antibacterial, antiviral, anti-inflammatory and anticancer (Jogendra et al. 2012). There are reports on appreciable (86 %) cytotoxic activity of the chloroform fractionate of methanol extract of *A*. *sessilis* at maximum concentration (100 µg/mL) (Chan et al. 2008). Alternanthin B, a flavonoid isolated from *Alternanthera philoxeroides* species is known to possess antitumor activity (Zhou et al. 1988). Ragasa et al. (2002) reported that the chloroform extract of the air-dried leaves of *A. sessilis* afforded a mixture of diastereomers of new ionone derivatives which are anti-proliferative agents. The HPLC of ethanolic extract of *A. sessilis* evidences ellagic acid to be a prominent chemical constituent. Ellagic acid possesses antiproliferative activity.

### Mode of action of drugs on cancer cells

A thorough review of literature embarks the different mechanisms of drug action on cancer cells. Interference of the electron transport mechanism of the cell by silver ions inhibits the respiratory mechanism as the silver cation readily oxidizes ATP resulting in the formation of silver (0). The replication and protein encoding of the bacteria get disrupted by the binding capability of silver to the DNA and RNA. The bacterial cell disruption aided by silver is similar to that of the action of platinum complex anticancer drug, cisplatin (Youngs et al. 2012).

Cisplatin, an anticancer drug has been commercially developed for selective killing of cancerous cells without any effect on non-cancerous or normal cells. Drugs used in cancer therapy enhance permeability and retention (EPR) effect, passively targeting the leaky vasculature of the tumor cells, since the cancer cells do not have a strong vasculature system compared to that of normal cells. Hence, the penetration of the drugs is easier and finally kills the cancer cells, but sometimes it enters into the normal cells and cause toxic effects (Jaracz et al. 2005; Blanco et al. 2009; Luo and Prestwich 2002; Leamon and Reddy 2004; Salazar and Ratnam 2007; Yoo and Park 2004).

To overcome these facets, the drugs can be loaded with nanoparticles and targeted moieties on the surface which will act against the particular receptor without affecting the normal cells. Many receptors have been discovered for cancer drug targeting, the commonly used one is folic acid (Kim 2006). The aqueous extract of *Taxus baccata* synthesized silver nanoparticles revealed potent anticancer effects on MCF-7 cells with an IC50 value of 0.25 μg/mL by MTT assay (Kajani et al. 2014). AntiABCG2 monoclonal antibody combined with AgNPs and Vincristine provide an efficient, targeted therapeutic method for inhibiting myeloma growth in mice (Dou et al. 2013). MDA-MB-231 breast cancer cells exposed to *Ganoderma neojaponicum Imazeki*-AgNPs after 24 h showed increased production, reactive oxygen species and hydroxyl radical. The apoptotic effects of AgNPs were further confirmed by the activation of caspase 3 and DNA nuclear fragmentation (Gurunathan et al. 2013a, b). The cytotoxicity of *T. divaricata* leaf extract synthesized AgNPs against human breast cancer cell indicates the presence of significant amounts of reducing entities (Devaraj et al. 2014).

Enhanced cytotoxic activity was observed for MCF-7 than A549 cells due to the increased cytotoxicity, decreased viability and proliferation which result in apoptosis through induced programmed cell death by irradiated AgNPs (MfouoTynga et al. 2014). The differential response of breast cancer cells to AgNPs induced hyperthermia, which implies AgNPs to be effective photothermal agents (Thompson et al. 2014). Apoptosis could be activated through Bax/BCl_2_ and caspase cascade mediated mitochondrial dysfunction, which potentially inhibits the proliferation of MCF-7 cells (Jeyaraj et al. 2015).

### Proposed mode of action of nanosilver on MCF-7 cells

**Fig. 6:**
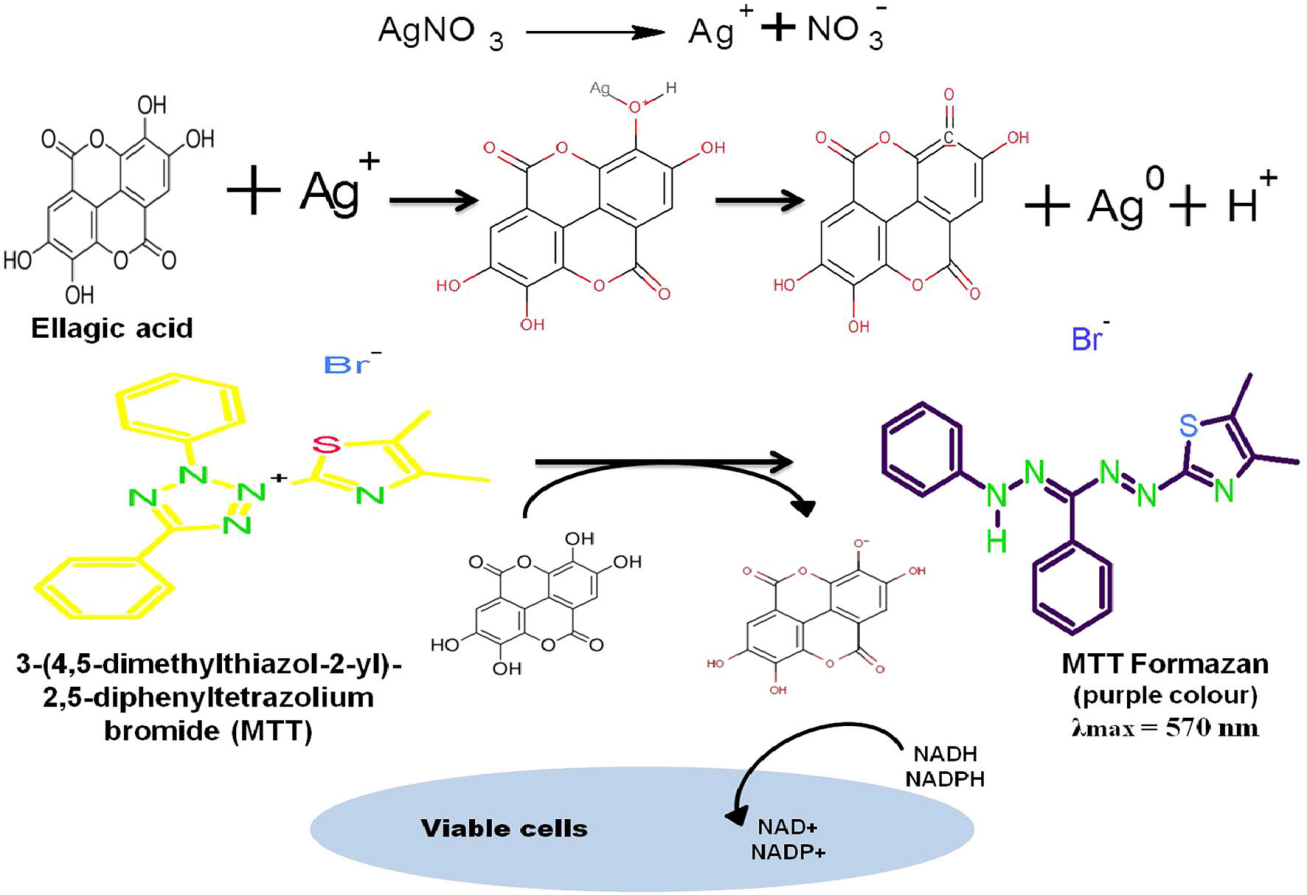
Proposed mode of action of ellagic acid-embedded silver nanoparticles on MCF-7 cell line

Figure 6 is the proposed mode of action of biogenically synthesized nanosilver embedded with ellagic acid on breast cancer cell lines. The nature of plant extract directly affects the physical, chemical and cytotoxic properties of the nanoparticles due to the interaction of nanoparticles with cells and intracellular macromolecules like proteins and DNA. Cellular uptake of nanoparticles leads to generation of reactive oxygen species which provoke oxidative stress. Cell damage by silver nanoparticles may be due to loss of cell membrane integrity, apoptosis and oxidative stress. *A.sessilis* is rich in beta ionones and flavonoid Alternanthin B the structures of which are given below. Molecules with ionone rings and Alternanthin B are known anti-tumor agents, though the mechanism of action is not well-defined in literature.

β-Ionone is an end-ring analog of β-carotenoid which possesses potent antiproliferative activity. Mo and Elson (2004) demonstrated that β-ionone and a variety of isoprenoids can inhibit the growth of malignant cells in different experimental models. The cell cycle was arrested in the human gastric adenocarcinoma cancer cell lines treated with β-ionone by inhibition of cell growth and DNA synthesis in a dose-dependent manner and this may be regulated by mitogen-activated protein kinase pathways (Liu et al. 2004; Dong et al. 2013). The impaired activity of cyclin-dependent kinase (CDK) 2 and decreased expression of positive regulators of G1 to S phase progression aided by mevalonate depletion results in a G1 phase cell cycle arrest. The inhibition of HMG-CoA reductase activity also mediates the depletion of mevalonate which contributes to the cell cycle inhibitory and anti-proliferative effects of β-ionone on human breast cancer cells (MCF-7) (Duncan et al. 2004).

The flavonoid rings and ionone rings might have interfered with the gene expression, immune modulation and would have boosted the antioxidant effect resulting in apoptosis and death of cancer cells. Further studies are needed to optimize the mechanism of AgNPs on cancer cells. 

The mechanism of ellagic acid on MCF-7 cell lines is reported. The inhibition of proliferation of MCF-7 breast cancer cells is through the modulation of the TGF-β/Smad3 pathway associated with decreased phosphorylation of RB proteins (Zhang et al. 2014). Blueberry extracts, rich in ellagic acid, modulate the PI3K/AKT/NF kappa B pathway, and inhibits the growth and metastasis of MDA-MB-231 breast cancer cells (Adams et al. 2010). Treatment with ellagic acid arrests G0/G1 cell cycle and induces apoptosis in bladder cancer T24 cells (Li et al. 2005). It also induces apoptosis through G1/S cell cycle arrest in SW480 colon cancer cells (Narayanan and Re 2001). Han et al. (2006) reported ellagic acid to significantly reduce HOS cell proliferation and induce apoptosis as evidenced by chromosomal DNA degradation and through the upregulation of Bax and activation of caspase-3.

Ellagic acid metabolizes to urolithins-A in the gut, which exerts a remarkable antiproliferative activity in human colon cancer cells (Caco-2, SW-480 and HT-29) (González-Sarrías et al. 2005). The treatment of ellagic acid with PC3 cells results in a dose-dependent inhibition of cell growth/cell viability accompanied by induction of apoptosis and cleavage of poly(ADP-ribose) polymerase (PARP) and morphological changes. Ellagic acid induces apoptosis by upregulation of c-fos and pS2 protein in MCF-7; whereas, it follows intrinsic pathway in MDA-MB-231 cells. Hence, ellagic acid may have different pathways or antiproliferative activity on human breast cancer cell lines (Kim et al. 2009).

It has also been shown previously that NPs interfere with the MTT assay by adsorbing the tetrazolium salt (Wörle-Knirsch et al. 2006), or by releasing metal ions which modify the catalytic activity of the mitochondrial reductases (Kroll et al. 2009). Gurunathan et al. (2013a, b) revealed that the potential cytotoxic effect of biologically synthesized AgNPs in MDA-MB-231 cells accompanied by inhibiting the growth of cells, concentration-dependent activation of LDH, increased level of ROS generation and activation of caspase-3 are considered to be the most significant of the executioner caspases resulting in cellular apoptosis.

The mode of action of AgNPs and ellagic acid portrays AgNPs embedded with ellagic acid to inhibit the proliferation of MCF-7 cells through Bax/BCl_2_ and caspase cascade-mediated mitochondrial dysfunction accompanied by the TGF-β/Smad3 pathway. The combination of these pathways may be the reason for the increased rate of cell inhibition even at lower concentration. Thus, the AgNPs fabricated ellagic acid exhibits 99 % anti-proliferation at a concentration of 25 µL/mL. The present result was found to be valid on comparing with that of the cell inhibition acquired using individual components viz, ellagic acid and AgNPs.

## Conclusion

Plant-mediated synthesis of silver nanoparticles using the extract of *A. sessilis* by sonication method advocates green nanotechnology. The results of the present cytotoxic study against human breast cancer MCF-7 cell lines by MTT assay revealed that silver nanoparticles serve as a potential anticancer drug compared to the standard cisplatin. Nanosilver showed excellent apoptosis rate due to their smaller size and spherical morphology. The present study contributes a novel and alternate approach in cancer therapy. The novelty of the present research is that complete cell inhibition (99 %) of breast cancer cell lines with plant-mediated nanosilver particles is obtained with 25 µg/mL, whereas it is 30 µg/mL for cisplatin, the standard drug used in breast cancer. Use of this edible plant extract eliminates the need to remove toxic byproducts during the formation of silver nanoparticles. Further studies are needed to optimize the mechanism of AgNPs on cancer cells.
